# Quality and quantity of sleep in multipatient versus single-room ICUs

**DOI:** 10.1186/cc10928

**Published:** 2012-03-20

**Authors:** M Van Eijk, A Slooter

**Affiliations:** 1 University Medical Center Utrecht, the Netherlands

## Introduction

Sleep fragmentation and deprivation is common in ICU patients [1]. It is assumed that the ICU environment (overexposure to sound and light during night-time) leads to disturbed sleep [2]. In our hospital, a new ICU was built with quiet, single-patient rooms with much daylight. This created an opportunity to study the effects of nursing environment on sleep quality and quantity in ICU patients.

## Methods

**Figure 1:**
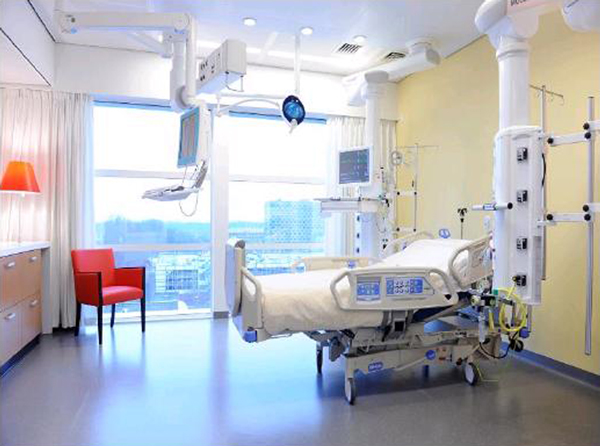
**New, single-room ICU**.

We included 21 postcardiothoracic surgery patients: 11 subjects were admitted to the old, ward-like ICU, and 10 patients to the new, single-room ICU (see Figure 1). Hypnograms were derived from a polysomnography from 07:00 p.m. to 07:00 a.m.

## Results

Both groups did not differ with respect to age, duration of surgery or use of psychoactive medication. Polysomnography recordings showed no differences in total sleep time and awakenings (63 ± 26 in the old ICU and 56 ± 30 in the new ICU). The mean percentage of sleep stages in the old versus new situation did not essentially different either: N1: 12.9% versus 8.0%, *P *= 0.21, ANOVA; N2: 80.3% versus 87.2%, *P *= 0.07, ANOVA; N3: 5.2% versus 2.5%, *P *= 0.18, ANOVA. Only REM sleep latency was longer in the old ICU: 314.7 versus 633.5 minutes, *P *= 0.02, ANOVA.

## Conclusion

Except for REM onset latency, sleep improvement was not achieved by changing a ward-like into a single-patient-room ICU environment. When striving for more natural sleep, attitudes towards nursing and medication may play a more important role than ICU design.
